# Growth and descent of the testes in infants with hypogonadotropic hypogonadism receiving subcutaneous gonadotropin infusion

**DOI:** 10.1186/s13633-016-0031-9

**Published:** 2016-07-04

**Authors:** Anne-Sophie Lambert, Pierre Bougneres

**Affiliations:** Department of Pediatric Endocrinology, Pôle I3E, Hôpital Bicêtre, Assistance Publique des Hôpitaux de Paris, Paris Sud University, Le Kremlin-Bicetre, France

**Keywords:** Gonadotropin infusion, Testes descent, Congenital Hypogonadotropic Hypogonadism, Infancy

## Abstract

**Background:**

One third of infants with congenital hypogonadotropic hypogonadism (CHH) are said to have micropenis and/or bilateral or unilateral cryptorchidism leading many of them to orchiopexy. Our previous study in two patients suggests that prolonged subcutaneous infusion of large doses of gonadotropins might normalize testicular function and growth.

**Case presentation:**

To confirm the effects of early and prolonged subcutaneous infusion of large doses of gonadotropins on growth and descent of the testes. Eight boys with CHH, aged 0.25–11 months. Testes were non-palpable in 5 or in high scrotal position in 3. CHH was isolated in 5 infants and part of a syndrome of combined pituitary hormonal deficits in the 3 others. In response to gonadotropin infusion, mean levels of testicular hormones were normalized. Complete testis descent occurred in 6 patients. Partial descent occurred in 2. Testes re-ascended in 1 patient. Testes and penis gained normal dimensions in all cases.

**Conclusion:**

Subcutaneous gonadotropin infusion seems able to induce testis descent in a large proportion of infants with CHH. If confirmed, this may allow patients to avoid testes surgery but studies in larger series are needed to evaluate the benefits of this treatment versus traditional orchiopexy.

**Electronic supplementary material:**

The online version of this article (doi:10.1186/s13633-016-0031-9) contains supplementary material, which is available to authorized users.

## Background

Hypogonadotropic hypogonadism (HH) is observed at birth in infants who have combined pituitary hormone deficits (CPHD) or isolated hypogonadotropic hypogonadism (IHH), two separate entities that have separate and multiple genetic etiologies. Cryptorchid or maldescended testes and small testicular volume are clinical hallmarks of congenital hypogonadotropic hypogonadism (CHH) [1]. We carried out a Pubmed search with the keywords “cryptorchidism”, “cryptorchid testes”, “testes descent”, “CHH”, “CPHD”, “hypopituitarism”, “Kallman”, “isolated HH or idiopathic HH”. Unexpectedly, this search retrieved only two studies allowing an estimation of the prevalence of cryptorchidism in patients with HH. These two studies were carried out in the same group of patients with IHH [2, 3]. Cryptorchidism was reported in one third of these patients and was bilateral in half of them or unilateral in the other half [2, 3]. Cryptorchidism, particularly if bilateral, is considered to have additional negative effects on the future fertility of patients with IHH [2].

A number of studies have examined the effect of human chorionic gonadotropin (hCG) or recombinant human LH (rhLH) and FSH (rhFSH) injections upon testicular descent in common cryptorchidism [4, 5]. Hormonal treatment is associated with testicular descent in some of these children, but rates generally do not exceed those seen with placebo by more than 10 % [6]. Surprisingly, a literature search found no comparable study in infants with CHH, in whom hCG was largely used to test testosterone secretion [1], but not for therapeutic purposes. In the absence of a systematic study of hCG treatment upon testes descent in infants with either IHH or combined pituitary deficits, the data collected in common cryptorchidism are often used instead, but this extrapolation may not be justified. We know of only two studies, totaling 3 infants with CHH, that have used gonadotropins in a therapeutic perspective. In the unique patient treated by Main et al., six months of twice-weekly subcutaneous injections of rhLH (20 IU) and rhFSH (21.3 IU) allowed a limited growth of the penis from 1.6 to 2.4 cm and a 1.7-fold increase of testes volume [7]. In the two patients treated by Bougnères et al., subcutaneous infusion of rhLH and rhFSH at much higher daily doses of 50 IU and 100 IU were used to mimic mini-puberty in two infants with CHH [8]. This treatment induced a tripling of penile length and a 4-fold increase in testes volume.

The other hormonal treatment that is classically used in infants with CHH is depot esters of testosterone [9]. Testosterone can be administered im, locally by a testosterone lotion or gel, transdermally by a testosterone patch or suppository. This treatment induces durable phallic growth but has no effect on testicular volume or descent. The lack of effect of testosterone alone upon testis descent contrasts with the classical hypothesis that the inguinoscrotal phase of testis descent, which takes place between the 25th and the 35th week of gestation but often occurs after birth in humans, is androgen-mediated and is directly affected by the lack of testosterone production in fetuses with CHH [10].

The usual treatment of maldescended testes is surgical orchiopexy. For children with common, so-called “idiopathic” cryptorchidism, recommendations advocate orchiopexy at 6–12 months [11]. These recommandations contrasts with the report collected by Pitteloud et al. in patients with IHH [3]. This report indicates that surgical repair was performed in 86 % of patients with cryptorchidism only between 4 and 35 years of age, at a mean age of 13 ± 2 years [3]. We have not been able to find a publication that has specifically examined or reviewed the indication, prevalence and long term results of orchiopexy in infants or children with CHH and cryptorchidism, whether they have IHH, including Kallman syndromes, or CPHD. This is even more surprising considering the fact that the surgical correction of cryptorchidism is advocated as a possible means for increasing the likelihood of future fertility [12, 13]. Anecdotical reports of orchiopexy were found in a number of publications following a Pubmed search using keywords such as “orchiopexy”, “CPHD”, “hypopituitarism”, “IHH”, “Kallmann”. See detailed comments and preferences in Additional file 1. These publications seems to indicate that orchiopexy is often used for the treatment of cryptorchidism in children with various genetic etiologies of CHH, and that in most cases, orchiopexy is performed at age 4 years or later, confirming the observation of Pitteloud et al. [3]. The treatment of cryptorchidism thus remains a yet-to-be explored clinical field of research aiming at the preservation of fertility potential in patients affected with CHH. The current study focuses on the effects of subcutaneous continuous recombinant LH and FSH infusion on the mal-descended testes in neonates with CHH, with the idea of proposing this treatment as an alternative to orchiopexy.

## Case presentation

Eight patients aged 6.03 ± 3.75 months (0.25–11 months) had CHH due to CPHD (*N* = 3) or IHH (*N* = 5). All infants had normal birth height and weight. All 8 patients had serum testosterone below 0.3 ng/ml (to convert into SI units, multiply by 3.47, ie 1 nmol /L) and LH <0.8 U/l during the time of physiological mini-puberty. The etiology of IHH was *KAL1* mutations in two patients, *KAL2* mutations in one patient, *SOX2* mutation in one patient; one patient with IHH remained without molecular diagnosis. The 3 patients with CPHD had growth hormone, TSH, ACTH deficiencies and ectopic neuro-hypophysis. We have used the classification of cryptorchidism proposed by Scorer [14], where testis location is defined as normal, high scrotal, supra-scrotal, inguinal or non-palpable [15]. In our study, 5/8 patients had non-palpable testes and 3/8 had palpable testes in a high non-scrotal position. Penile length was measured by the method of Schonfeld [16] the penis is stretched to resistance, and length is measured along the dorsal aspect from the pubis to the tip of the glans. Volume of the testes was carefully determined through palpation and direct measurement. Clinical characteristics of the patients are described in Table 1.

**Table 1:** Characteristics of the 8 patients with CHH treated by rhLH and rh FSH infusion

Patients	Classification	Age at onset of treatment (months)	Before treatment		After treatment				
			Testes position	Penile length (mm)	Time to descent (months)	Testes volume (ml)	Testes position	Penile length (mm)	Duration of treatment (months)
P1	CPHD	6	Non palpable	18	1	0.61	High scrotal	30	6
P2	CPHD	11	Non palpable	20	4	2	Intra-scrotal	33	6
P3	CPHD	10	High-scrotal	30	1	2,1	Intra-scrotal	70	6
P4	IHH	4.5	Non palpable	21	4	0.76	Intra-scrotal	30	6.5
P5	IHH	2.5	Non palpable	20	6	0.7	High-scrotal Intra-scrotal	35	6.5
P6	IHH	9	Non palpable	19	4	1,16	Intra-scrotal	40	5
P7	IHH	5	High-scrotal	28	4	0,89	Intra-scrotal	52	5
P8	IHH	0.25	High-scrotal	13	5	1,95	Intra-scrotal	30	6

*IHH* isolated hypogonadotropic hypogonadism, *CPHD* Combined Pituitary Hormonal Deficits (CPHD)

Following the favorable preliminary observations made in 2008 [8], we have since then routinely applied the subcutaneous infusion of rhLH and rhFSH to all infants with CHH referred in our department. The treatment started after explanations were given to the parents and consent was obtained. Ethical approval was obtained for the previous study from our local St Vincent Pediatric Ethical Committee (SVP-2007-04). rhLH (Luveris 75 IU®) and rhFSH (Gonal-F 75 IU®) were obtained from Serono (Geneva, Switzerland) and dissolved in solvent for infusion. A Paradigm insulin pump (Minimed, Northridge, CA) whose catheter was changed every 48–72 h was used for infusion. The biological stability of the infused rhLH and rhFSH within the pump system was ascertained by the stability of the plasma levels of LH, FSH, testosterone and inhibin B (INB) in the first two infants [8] when sampled at various times such as 12 h and 72 h, the levels of these hormones were found to be steady. Continuous infusion of rhLH and rhFSH was administered at a daily rate of 50 and 75–150 IU, respectively. These doses were used because we had previously observed that they allowed Leydig and Sertoli cell hormones to reach the normal range during minipuberty [17]. Indeed, daily infusion of 50 IU rhLH over 20 weeks induced LH levels of 4.8 ± 2.4 IU/ml and testosterone levels at 1.5 ± 0.1 ng/ml in the two studied infants. For rhFSH infusion rates, we observed that high daily doses of 75–150 IU (resulting in FSH levels of 49 ± 17 IU/ml) were needed to obtain mean values of AMH and inhibin B (INB) levels of 563 ± 204 pmol/l and 717 ± 234 pg/ml. In the current report, we have not tested graded infusions nor did we carry out a systematic dose–response study. Instead we started with daily doses of 50 IU rhLH and 75 IU of rhFSH, then used an approximate dose adjustment aiming at maintaining hormone levels within the normal range of mini-puberty [18, 19]. No adverse effects were observed at the cutaneous infusion site in any of the infants.

Classical replacement therapy with growth hormone, L-thyroxine, and hydrocortisone was administered to the patients with CPHD.

Serum FSH, LH, AMH, INB and testosterone concentrations were measured using RIA in the same laboratory as reported [20]. One-sided paired *t* test was used to analyze the observed changes of penile length, testis volume and hormone levels.

Treatment duration was 6 ± 0.58 months. Follow-up after cessation of gonadotropin infusion was 35.5 months [13–54 months]. The continuous subcutaneous infusion of rhLH and rhFSH increased serum gonadotropins and testosterone, to values normally observed during mini-puberty (Table 2). However, serum AMH and inhibin B both failed to reach the normal mini-puberty range in 1/8 patients.

**Table 2:** Serum concentrations of hormones before and during treatment

	Before treatment					During treatment				
	FSH (UI/l)	LH (UI/l)	T^a^ (ng/ml)	AMH (pmol/l)	INB (pg/ml)	FSH (UI/l)	LH (UI/l)	T (ng/ml)	AMH (pmol/l)	INB (pg/ml)
Patients with CPHD										
P1	0.4	0	0.06	103	5	18	1.2	1	264	55
P2	0.03	<0.1	0.02	675	100	21	2.9	1.4	1644	505
P3	0.61	0.7	0.02	1984	155	18	2	2.8	1040	544
Patients with IHH										
P4	1	0.4	0.05	125	91	40	4.6	2.8	29	111
P5	0.8	0.1	0.04	595	73	24	4.7	3.3	565	401
P6	0.1	<0.1	0.05	250	5	41	5.4	3.86	666	287
P7	0.3	<0.1	0.02	665	64	32	4.6	3.9	668	514
P8	0.21	<0.1	0.07	270	14	59	9.5	9.6	890	530
Mean value	0.48	0.15	0.04	583	63.3	31^b^	4.4^b^	3.6^b^	721	368
Normal values^c^	0,2–4	0,5–7,1	0.52–4.8	251–679	254–513	0,2–4	0,5–7,1	0.5–4.8	251–679	254–513

*IHH* isolated gonadotropin deficiency, *CPHD* Combined Pituitary Hormonal Deficits (CPHD), *INB* inhibin B

*T* testosterone

^a^To convert T into SI unit (nmol/l), multiply by 3.47

^b^ *p* < 0.005 vs baseline values

^c^Range of normal values taken from reference [18, 19]

Mean stretched penile length increased from 20.2 mm [13–28 mm] to 37.4 mm [30–52 mm] (*p* < 0.001) in infants with IHH and from 22.6 mm [18–30 mm] to 44.3 mm [30–70 mm] (*p* < 0.001) in infants with CPHD. Mean final testes volume was 1.27 ml [0.61–2.1 ml] Simultaneously, testicular volume increased from 0.43 ml [0.1–0.68 ml] to 1.64 ml [0.33–2.1 ml] (*p* < 0.001) in the 3 children who had testes palpable in high scrotal position. The details of testis descent are shown in Table 2. Among the 5 patients having their testes in abdominal position, bilateral descent to the bottom of the scrotum occurred in three (P2, P4 and P6) and bilateral incomplete descent in high scrotal position occurred in one (P1). One of the 5 patients with abdominal testes (P5) had a descent of his left testis to the bottom of the scrotum, while his right testis remained in high scrotal position. In total, among the 10 testes that were initially located in abdominal position, complete descent occurred in 7, and incomplete descent in 3. The three patients who had testes initially palpable in high scrotal position showed complete scrotal descent during treatment. In one case (P4), however, both testes re-ascended 11 months after their descent, thus underwent orchiopexy. The cost of rhLH and rhFSH infusions per studied child over the 6 reported months averages 18,000 USD.

## Discussion

A satisfactory sex life and fertility are the two main objectives of the treatment of CHH. To allow for fertility, therapeutic regimens proposed in adulthood used the administration of hCG/HMG or hCG/rhFSH [21–25] or pulsatile GnRH with or without rhFSH in adolescents [26, 27]. Cryptorchidism is estimated to affect approximately one third of patients with IHH [2, 3] and an important, but unknown proportion of infants with CPHD.

Patients affected with CHH who have cryptorchid testes have decreased hormonal response to gonadotropins at adolescence [26] or a less favourable fertility outcome [2] than those in whom spontaneous testis descent occurred at times of fetal or neonatal life. This may be due to intrinsic anomalies of the undescended testes, an acquired aggravation of testes abnormalities due to their prolonged intra-abdominal location [28], or could be secondary to lesions generated by surgical procedure needed to move an undescended testis into the scrotum, which carries a greater risk of damage to the testis when the testis is very small as in all infants with CHH.

It is noteworthy in this respect that many of the studies reporting treatment outcomes in male patients with CHH were biased by the exclusion of patients with cryptorchidism [22, 24, 29–31]. The good therapeutic results that have been published regarding gonadotropin or GnRH treatment of CHH are thus likely to be less favorable if cryptorchid patients are included, as observed by Pitteloud et al. [2].

Boys with CHH lack all mini-pubertal hormonal events [32]. A potential contributor for azoospermia and infertility in adult men is the deficient proliferation of immature Sertoli cells in infancy due to FSH insufficiency. The artificial increase of Sertoli cell activity during early infancy might have beneficial effects on gonadal development, proliferation of immature Sertoli cells and spermatogonia that will help fertility in adult life, but this is entirely speculative. The current report on the effects of rhLH and rhFSH confirms our previous observation of correction of Sertoli and Leydig hormone pattern, and growth of phallus length and testes volume [8]. A new finding was the testicular descent observed in almost all treated patients. Despite the lack of an untreated group and the small number of studied infants, the current report suggests that early infusion of recombinant gonadotropins is able to trigger testes descent in children with CHH, even when testes are initially in abdominal position. This effect was seen as late as the 17 months of life and therefore does not seem to be confined to the time of the physiological mini-puberty. The descent of testes occurred within a mean of four months following the onset of gonadotropins infusion, which may be beneficial since a recent study showed a negative relationship between the duration of a supra-scrotal position and testicular growth [11]. In a few cases, the descent induced by gonadotropin infusion may be reversible, since testes re-ascended in one patient 11 months after their descent. Larger trials will be necessary to better define the window of efficacy of gonadotropin infusion for inducing testis descent.

Our choice of using large doses of rhFSH doses needs to be discussed. It was dictated by the previous observation in the first two reported infants [8], in whom we observed that daily dosages of 50 IU rhFSH per day resulted in circulating levels of AMH and inhibin B well below the normal range. We then decided to increase the daily doses to 75–150 IU to force Sertoli cells to produce AMH and inhibin B at a more physiological level, as observed in the current report. We speculated that in patients born with both HH and cryptorchidism, there may be some resistance of Sertoli cells to FSH, possibly because of the prolonged lack of exposure to pituitary FSH during prenatal life.

Another aspect to be discussed in the current report is the deleterious effect that high circulating gonadotropin could have on germ cells in cryptorchid testes. Such deleterious effect have only been reported in boys with idiopathic cryptorchidism (with or without primary testicular insufficiency) treated with hCG, not in the CHH population, or in response to rhLH or rhFSH [33, 34].

## Conclusion

The early continuous infusion of rhLH and rhFSH during the first months of life seems to allow the descent and growth of a large proportion of cryptorchid testes in infants with CHH, as late as the 11th month of life. These preliminary observations raise several questions. Which of the two gonadotropins is responsible for the testis descent? Should this treatment replace the traditional orchiopexy in children with documented CHH? Would repeated hCG injections allow comparable effects at a lower cost? hCG is recognized as of limited efficacy in infants with common cryptorchidism. It is thus possible, and even likely, that repeated hCG injections would prove of greater efficacy when cryptorchidism is related to gonadotrophin deficiency. Owing to its simplicity and low cost, this approach, which has not been studied previously in infants with HH, will need a comparative evaluation in the future, both with orchiopexy and with the rhLH/rhFSH pump approach. Will the future fertility benefit from both the early descent of the testes and the stimulation of Sertoli cell function and/or proliferation induced by the rhLH and rhFSH infusion? Only prolonged follow-up of infants and large international trials will hopefully provide answers to the yet-to-be solved problem of the early management of cryptorchidism in CHH. In this regard, a possible merit of the current report is to bring the case of cryptorchidism associated with CHH in the frontline of future therapeutic approaches for preserving fertility potential in these patients.

## Abbreviations

CHH, Congenital Hypogonadotropic Hypogonadism; HH, Hypogonadotropic Hypogonadism; CPHD, Combined Pituitary Hormonal Deficits; IHH, Isolated gonadotropin deficiency; rh, Recombinant; T, Testosterone; INB, Inhibin B

## Additional file

Additional file 1: Specific bibliography of orchiopexy in hypogonadotropic hypogonadism patients. (DOCX 147 kb)
