# Supplementary material for: Growth and descent of the testes in infants with hypogonadotropic hypogonadism receiving subcutaneous gonadotropin infusion
Source: Int J Pediatr Endocrinol. 2016 Jul 4;2016:13. doi: 10.1186/s13633-016-0031-9 (PMC4931699; doi:10.1186/s13633-016-0031-9)
Supplement: Additional file 1: — Specific bibliography of orchiopexy in hypogonadotropic hypogonadism patients. (DOCX 147 kb) [file 13633_2016_31_MOESM1_ESM.docx]

**Supplemental S1**

A number of publications in patients with CHH, whether they have CPHD or IHH, do not mention testes position nor orchiopexy. Our pubmed search retrieved a few publications motivated by genetic information, that indicated the occurrence and timing of orchiopexy in patients with IHH (1)(2)(3)(4)(5)(6)(7)(8)(9)(10)(11)(12). We have not been able to find comparable information for patients with CPHD.

1. Bhangoo APS, Hunter CS, Savage JJ, Anhalt H, Pavlakis S, Walvoord EC, et al. Clinical case seminar: a novel LHX3 mutation presenting as combined pituitary hormonal deficiency. J Clin Endocrinol Metab. 2006;91(3):747‑53.

2. Izumi Y, Suzuki E, Kanzaki S, Yatsuga S, Kinjo S, Igarashi M, et al. Genome-wide copy number analysis and systematic mutation screening in 58 patients with hypogonadotropic hypogonadism. Fertil Steril. 2014;102(4):1130‑6.e3.

3. Reynaud R, Barlier A, Vallette-Kasic S, Saveanu A, Guillet M-P, Simonin G, et al. An uncommon phenotype with familial central hypogonadism caused by a novel PROP1 gene mutant truncated in the transactivation domain. J Clin Endocrinol Metab. 2005;90(8):4880‑7.

4. Vieira TC, Dias da Silva MR, Cerutti JM, Brunner E, Borges M, Arnaldi LT, et al. Familial combined pituitary hormone deficiency due to a novel mutation R99Q in the hot spot region of Prophet of Pit-1 presenting as constitutional growth delay. J Clin Endocrinol Metab. 2003;88(1):38‑44.

5. Tenenbaum-Rakover Y, Sobrier M-L, Amselem S. A novel POU1F1 mutation (p.Thr168IlefsX7) associated with an early and severe form of combined pituitary hormone deficiency: functional analysis and follow-up from infancy to adulthood. Clin Endocrinol (Oxf). 2011;75(2):214‑9.

6. Dissaneevate P, Warne GL, Zacharin MR. Clinical evaluation in isolated hypogonadotrophic hypogonadism (Kallmann syndrome). J Pediatr Endocrinol Metab JPEM. 1998;11(5):631‑8.

7. Chan E, Wayne C, Nasr A, FRCSC for Canadian Association of Pediatric Surgeon Evidence-Based Resource. Ideal timing of orchiopexy: a systematic review. Pediatr Surg Int. 2014;30(1):87‑97.

8. Semple RK, Achermann JC, Ellery J, Farooqi IS, Karet FE, Stanhope RG, et al. Two novel missense mutations in g protein-coupled receptor 54 in a patient with hypogonadotropic hypogonadism. J Clin Endocrinol Metab. 2005;90(3):1849‑55.

9. Gonçalves C, Bastos M, Pignatelli D, Borges T, Aragüés JM, Fonseca F, et al. Novel FGFR1 mutations in Kallmann syndrome and normosmic idiopathic hypogonadotropic hypogonadism: evidence for the involvement of an alternatively spliced isoform. Fertil Steril. 2015;104(5):1261‑7.e1.

10. Mengen E, Tunc S, Kotan LD, Nalbantoglu O, Demir K, Gurbuz F, et al. Complete Idiopathic Hypogonadotropic Hypogonadism due to Homozygous GNRH1 Mutations in the Mutational Hot Spots in the Region Encoding the Decapeptide. Horm Res Paediatr. 2015;

11. Demirbilek H, Ozbek MN, Demir K, Kotan LD, Cesur Y, Dogan M, et al. Normosmic idiopathic hypogonadotropic hypogonadism due to a novel homozygous nonsense c.C969A (p.Y323X) mutation in the KISS1R gene in three unrelated families. Clin Endocrinol (Oxf). 2015;82(3):429‑38.

12. Hu Y, Bouloux P-M. X-linked GnRH deficiency: role of KAL-1 mutations in GnRH deficiency. Mol Cell Endocrinol. 2011;346(1-2):13‑20.
